# Computational design of anti-cancer peptides tailored to target specific tumor markers

**DOI:** 10.1186/s13065-024-01143-0

**Published:** 2024-02-22

**Authors:** Aisha Naeem, Nighat Noureen, Shaikha Khalid Al-Naemi, Jawaher Ahmed Al-Emadi, Muhammad Jawad Khan

**Affiliations:** 1 QU Health, Qatar University, P.O. Box 2713, Doha, Qatar; 2 Cancer Center and Department of Pediatrics, School of Medicine, Texas Tech University Health Sciences Center School of Medicine, Lubbock, TX 79430 USA; 3 Department of Biomedical Sciences, Qatar University, P.O. Box 2713, Doha, Qatar; 4 Department of Biosciences, COMSATS University Islamabad, Islamabad, 45550 Pakistan

**Keywords:** Anticancer peptides, Homology modeling, CXCR1, DcR3, OPG

## Abstract

Anti-cancer peptides (ACPs) are short peptides known for their ability to inhibit tumor cell proliferation, migration, and the formation of tumor blood vessels. In this study, we designed ACPs to target receptors often overexpressed in cancer using a systematic in silico approach. Three target receptors (CXCR1, DcR3, and OPG) were selected for their significant roles in cancer pathogenesis and tumor cell proliferation. Our peptide design strategy involved identifying interacting residues (IR) of these receptors, with their natural ligands serving as a reference for designing peptides specific to each receptor. The natural ligands of these receptors, including IL8 for CXCR1, TL1A for DcR3, and RANKL for OPG, were identified from the literature. Using the identified interacting residues (IR), we generated a peptide library through simple permutation and predicted the structure of each peptide. All peptides were analyzed using the web-based prediction server for Anticancer peptides, AntiCP. Docking simulations were then conducted to analyze the binding efficiencies of peptides with their respective target receptors, using VEGA ZZ and Chimera for interaction analysis. Our analysis identified HPKFIKELR as the interacting residues (IR) of CXCR-IL8. For DcR3, we utilized three domains from TL1A (TDSYPEP, TKEDKTF, LGLAFTK) as templates, along with two regions (SIKIPSS and PDQDATYP) from RANKL, to generate a library of peptide analogs. Subsequently, peptides for each receptor were shortlisted based on their predicted anticancer properties as determined by AntiCP and were subjected to docking analysis. After docking, peptides that exhibited the least binding energy were further analyzed for their detailed interaction with their respective receptors. Among these, peptides C9 (HPKFELY) and C7 (HPKFEWL) for CXCR1, peptides D6 (ADSYPQP) and D18 (AFSYPFP) for DcR3, and peptides P19 (PDTYPQDP) and p16 (PDQDATYP) for OPG, demonstrated the highest affinity and stronger interactions compared to the other peptides. Although in silico predictions indicated a favorable binding affinity of the designed peptides with target receptors, further experimental validation is essential to confirm their binding affinity, stability and pharmacokinetic characteristics.

**Supplementary Information:**

The online version contains supplementary material available at 10.1186/s13065-024-01143-0.

## Highlights

- An in silico strategy to design anti-cancer peptides with high affinity for differentially expressed receptors (targets) on cancer cells, specifically CXCR1, DcR3, and OPG receptors.
- Interacting residues of natural ligands and their receptors as a guide to creating a peptide library.
- AntiCP to assess the anti-cancer properties of the designed peptides.
- Docking simulations to analyze the binding efficiencies of the peptides with their respective target receptors.

**Supplementary Information:**

The online version contains supplementary material available at 10.1186/s13065-024-01143-0.

## Introduction

Traditional chemotherapy drugs, though effective, often come with debilitating side effects due to their lack of specificity and harming healthy cells during the treatment. Target specificity, selectivity and multidrug resistance remain among the most significant challenges in the ongoing battle against cancer. Peptide-based therapeutics offer a promising avenue for addressing these challenges in cancer treatment. Peptides, short chains of amino acids, are versatile and can be engineered to interact with specific tumor markers, offering higher target specificity and lower off-target effects [1]. Peptide-based drug discovery has explored numerous targets ranging from receptors to enzymes, with the potential to modulate intracellular signaling. Recent successes in peptide-based drugs have further expanded the range of potential therapeutic targets, providing researchers with a more diverse array of options to explore anticancer therapies [2, 3].

Despite the increased number of pharmacological formulations of the peptides, multifaceted challenges such as specificity, stability, delivery and bioavailability still exist. Numerous peptides have originated from animal and plant sources or are recombinant or synthetic peptides. However, peptides mimicking natural ligands that have been involved in cancer-related signaling pathways have received great attention and could overcome some of these problems. Generally, such peptides have demonstrated stable pharmacokinetic profiles, low toxicity, and minimal immunogenicity. However, ensuring peptide stability and efficient delivery to the target site under physiological conditions remains critical due to enzymatic degradation and proteolysis [4]. Moreover, the emergence of resistance by cancer cells over time poses a substantial clinical concern, underscoring the importance of developing peptides that retain efficacy over prolonged periods [5]. This necessitates a comprehensive, interdisciplinary approach, where ongoing explorations and modifications of anticancer peptides will play pivotal roles in both cancer prevention and treatment.

Undoubtedly, receptor-based peptide therapeutics have had the most profound impact in this field [6]. The primary focus revolves around targeting and inhibiting the uncontrolled proliferation of cells driven by overexpressed tumor proteins, particularly cell membrane receptors [7–9]. These peptides were strategically engineered to bind within the small, functionally critical cavities of the target proteins, disrupting specific catalytic centers or interfering with the binding sites of natural substrates. In silico peptide designing offers valuable resources for designing and prescreening peptides before their costly and labor-intensive in vivo synthesis, modifications and characterization [10–14].

In this study, peptide inhibitors were designed against three well-known tumor-specific receptors: CXC chemokine receptor type 1 (CXCR1) [15, 16], decoy receptor 3 (DcR3) [17] and osteoprotegerin (OPG) [18–20]. These proteins play crucial roles in inhibiting apoptosis directly or indirectly. CXCR1, in association with ligand IL8, controls the leukocyte transmission into tumor cells, modifies tumor immune response, regulates angiogenesis, increases tumor growth and survival, and promotes metastasis. DcR3 is an immunomodulator whose expression is elevated in tumors offsets the effect of TL1A and TRAIL and regulates the metastatic potential of cancer cells [21, 22]. OPG binds to RANKL to regulate bone metastasis, control tumor invasion in bone and modulate cellular integrity [20]. Briefly, our methodology involved generating a peptide library against CXCR1, OPG and DcR3, utilizing the information of interacting residues between the receptor and its corresponding ligand. Subsequently, the peptides were docked against their respective targets, and those demonstrating the lowest binding energies along with anticancer properties were identified as the top candidates. The objective is to establish a groundwork for developing precise and efficient anti-cancer therapies customized to the distinct molecular features of tumors, thereby enhancing the target specificity and pharmacokinetic profile of the peptides [13, 14].

## Methods

### Selection of target receptors

**Fig. 1:**
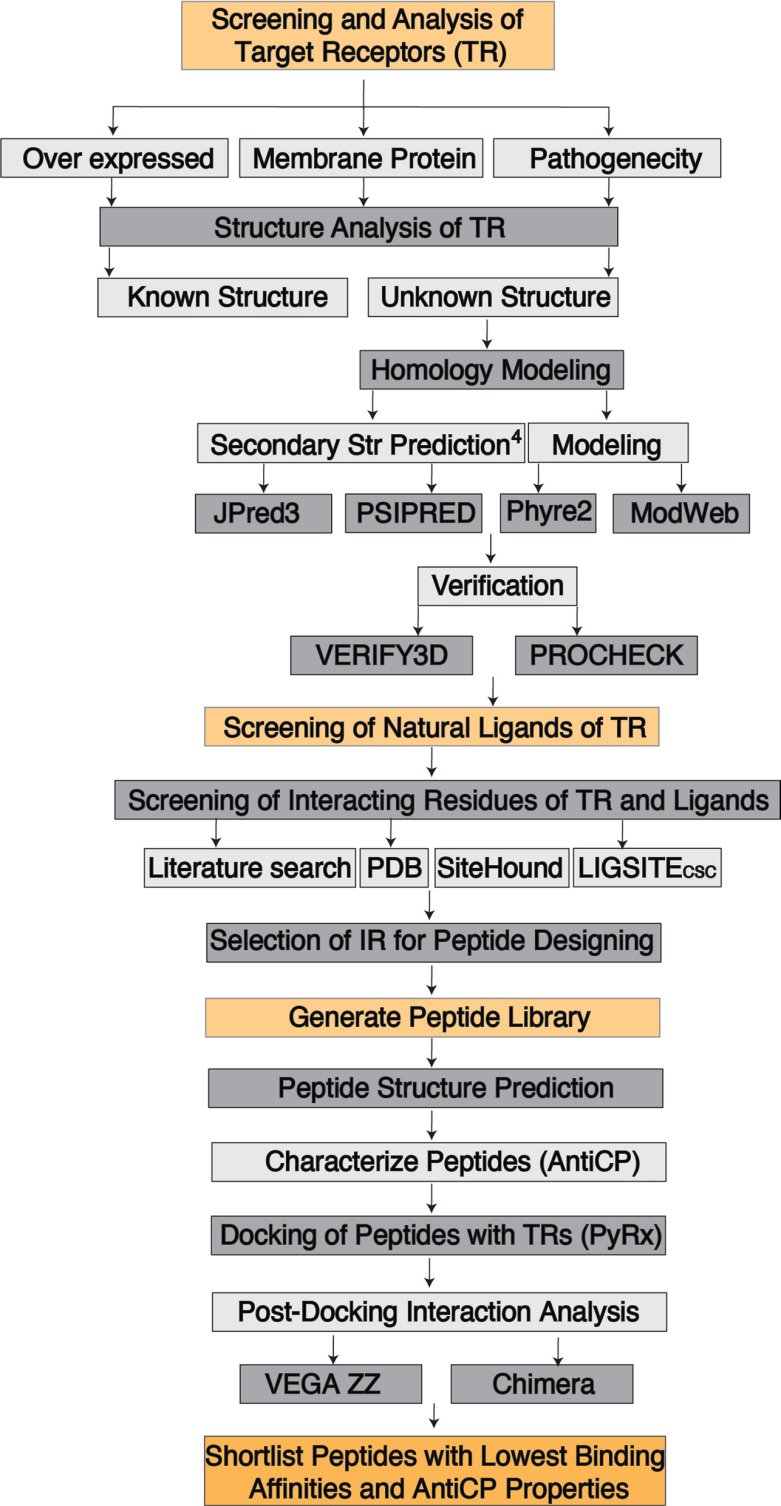
The workflow of the study. *TR* Target Receptors, *IR* Interacting Residues, *Str* Structure

The workflow of this study comprised several pivotal stages, primarily encompassing the identification of target receptors (TR), screening for their corresponding natural ligands, protein modeling, analysis of interacting residues (IR) between ligands and their TRs, and the generation and subsequent screening of a peptide library (Fig. 1).

The screening of TRs was based on their pathogenic role in cancer progression. The criteria for selection of TRs were overexpression in cancer, resistance to apoptosis, involvement in metastasis, invasion and support of angiogenesis for nutrient supply. The expression profiles of the selected protein were further validated in both normal and cancer cells from The Human Protein Atlas [23]. Based on the above parameters, three well-known tumor recepetor proteins i.e., CXCR1, DcR3 and OPG were shortlisted for downstream analysis. The specifics of the signaling pathway and the upstream and downstream regulators of these TRs were acquired from Target Explorer [24] (Table 1).

**Table 1:** Biological details of the selected receptor proteins

Targets	Cellular location	Type	Regulates	Regulated by	Signaling pathways	Natural Ligands
CXCR1	Cell membrane	Receptor	Ca2 + Erk1/2 CCR5 CXCR4	IL8 TNF Lipopoly- saccharides	IL8 Signaling Agranulocytes adhesion	IL8 CXCL6
OPG	Extracellular space	Decoy Receptor Secreted	RANKL RANK Ca^+2^ TRAIL DR4	IL1 RANKL TNF	NF-KB Signaling IL-6 Signaling	RANKL TRAIL APRIL RUNX2
DcR3	Extracellular space	Decoy Receptor Secreted	IL6, IL10, IL2, IFNG	VEGF MTOR	PI3K/AKT/GSK-3beta/beta-catenin signaling pathway	FASLG TNFSF14 TNFSF15 PPP1R7

### Protein structure of selected receptors

We obtained 3D structures of selected proteins from the Research Collaboratory for Structural Bioinformatics Protein Data Bank (RCSB PDB). If 3D crystal structures for the proteins were unavailable, computational modeling was performed using JPred [25] and PSIPRED [26]. The tertiary structure was obtained from ModWeb [27], CPH model 3.2 server [28] and Phyre2 [29]. Phyre2 was primarily used to identify a suitable template through pairwise sequence alignment, after which the structure was predicted based on that template. The modeled structure was verified using Verify 3D [30] and PROCHECK [31]. Structures obtained from various tools were highly consistent. The physiochemical properties of OPG proteins were computed using ProtParam [32]. ProtParam takes protein sequence as input and computes amino acid composition, the total number of amino acid residues in protein, molecular weight, total number of positive and negative charges on protein, atomic formula, total number of atoms, aliphatic index instability index and average hydropathicity (Additional file 1: Table S1) [32].

### Prediction of interacting residues (IR)

The IR of the natural ligands and their target proteins served as a basis for designing peptides against the TRs (Table 2). The amino acid residues of TR involved in interactions with their respective ligands were obtained from research articles, PDB and bioinformatics tools LIGSITE_CSC_ [33], Active Site Prediction (ASP) [34] and SiteHound [35]. The binding residues obtained from various sources were validated through result comparisons.

**Table 2:** Interacting residues of ligands and their corresponding target proteins for peptide design

TR-Ligands	Interacting residues
IL8-CXCR1	His18-Pro19-Lys20-Phe21-Ile22-Lys23-Glu24-Leu25-Arg26
TL1A-DcR3	Thr118-Asp119-Ser120-Tyr121-Pro122-Glu123-Pro124
	Thr172-Lys173-Glu174-Asp175-Lys176-Thr177-Phe178
	Leu56-Gly57-Leu58-Ala59-Phe60-Thr61-Lys62
RANKL-OPG	Ser246-Ile247-Lys248-Ile249-Pro250-Ser251-Ser252
	Pro300-Asp301-Gln302-Asp303-Ala304-Thr305-Tyr306-Pro307

The PDB file of the target was uploaded to LIGSITE_CSC_ with a grid spacing of 1.0 Å and a 5.0 Å probe radius to identify the binding site. Subsequently, the ASP server predicted cavities and assessed them based on the physiochemical properties of amino acids lining these cavities. It is worth noting that the top three predicted cavities have a reported accuracy of 92%. The PDB file was successfully processed, leading to the identification of ten binding cavities via the ASP online server. These binding sites were further identified using SiteHound, with CMET-Methyl Carbon serving as the probe. Finally, the IR of the target receptor proteins and ligands were visualized using Chimera.

### Peptide library

*Analog Generation:* Based on the interacting residues as identified above (Table 2), a library of peptide analogs from these sequences was generated through permutation using the online tool AntiCP [36]. The physiochemical properties of all the peptides were studied by AntiCP.

### Prediction of the tertiary structures of peptides

The prediction of the tertiary structures of peptides was carried out using Pepstr [37]. This prediction process encompassed three steps: (1) The forecast of secondary structures, including loops, alpha helices, and turns, using the beta-turn method; (2) Generating the initial conformation for the given sequence, with phi and psi values corresponding to the predicted secondary structures; (3) Subjecting the initial conformation to energy minimization and dynamic simulations. Subsequently, the final conformation was saved in the PDB file format [37].

### Molecular docking

The analogs were subjected to docking with their respective targets using PyRx v0.8 [38]. PyRx computed binding energies, identified interacting residues in both the TR and peptides and categorized the types of interactions between target receptors and peptides. The docking results obtained from PyRx were saved in pdbqt format. Subsequently, the out_pdbqt files of both the ligands and the proteins were processed in VEGA ZZ [39, 40]. The output files for both the protein and ligands were combined in VEGA ZZ and saved in pdb format. For a more comprehensive analysis binding energies, interacting residues of proteins and peptides, hydrogen bonding, hydrophobic interactions and other external bonds were also examined. This analysis was performed by opening the prepared.pdb file from VEGA ZZ [40] in LIGPLOT [41] which generated 2D plots illustrating the protein–ligand complexes.

### Cross-binding interactions with homologues of TR

Beyond our primary focus on the main TR, we also aimed to investigate the binding affinity of the designed peptides against other receptors that share high homology with the selected receptors. To explore these interactions, we conducted sequence comparisons between the TR and their homologs that share the same ligands using EMBOSS Needle and PSI-BLAST [42]. EMBOSS Needle facilitates the determination of optimal sequence alignments and reveals the degree of sequence identity between them. To gain deeper insights, we conducted a search for homologous structures of TR in the database using PSI-BLAST. Subsequently, the selected peptides were subjected to docking with the homologs of TRs, and the results of this docking were analyzed and compared with the docking results of the ligands with primary receptors.

## Results

**Fig. 2:**
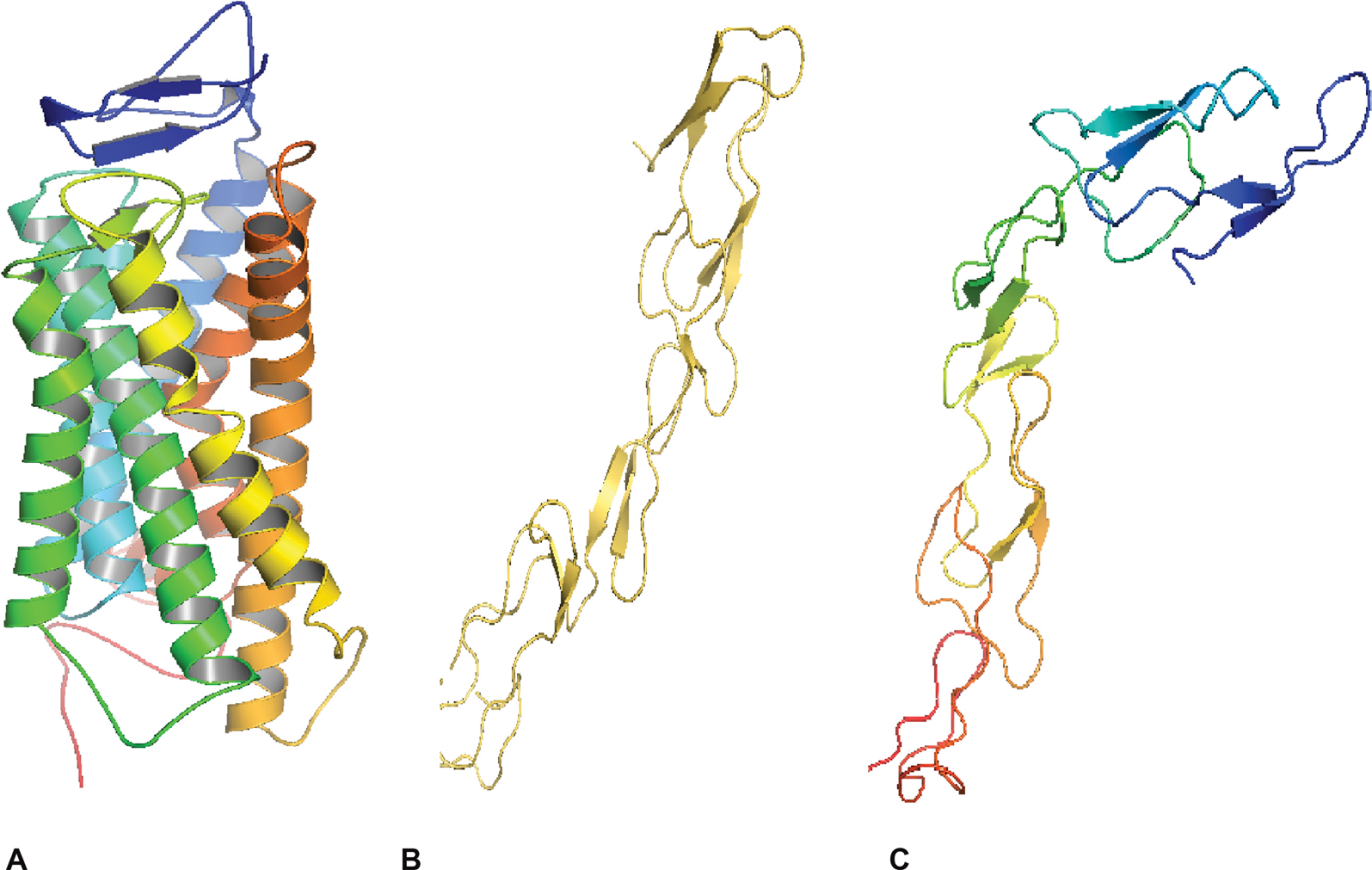
Crystal structures of target receptors: **A** CXCR1 (PDB ID 2LNL). It consists of 7 TM and N-terminus and C-terminus; **B** DcR3 (PDB ID 3MHD); **C** Predicted model of OPG using Phyre2. The segment 26–186 of OPG is modeled with 99.9% confidence using PDB ID 3urf as a template

The 3D structure files were retrieved from PDB for CXCR1 (PDB ID: 2LNL) and DcR3 (PDB ID: 3MHD) (Fig. 2A, B). The N-domain of CXCR1 was missing from the crystal structure so the N domain was modeled. The crystal structure of OPG was unknown so homology modeling was performed for OPG (Fig. 2C).

### Identification of interacting residues of target proteins

Interacting sites for all three receptors were systematically identified and verified through various methods including a comprehensive literature search, SiteHound, Active Site Prediction and LIGSITE_CSC._ The details of all identified IR residues are provided in Additional file 2.

*CXCR1*: The PDB complex structure with IL8 revealed binding regions at 17–24 and 9–14. The literature search highlighted the significance of the N domain (residues 1–39) and the extracellular loop (residues 97–111) in facilitating ligand binding (Additional file 1: Table S2) [43, 44]. The collective insights from these sources consistently pinpointed residues within the N-domain and extracellular loops as active binding sites for CXCR1 [45].

*DcR3*: The binding regions of DcR3 from PDB complexes with TL1A and LIGHT were identified to contain residues 72 to 251 and 29 to 198, respectively. The literature review identified critical amino acid residues crucial for binding, including Phe81, Trp84, Leu85, Arg89, Trp90, Gly96, Cys95, Asn92, Tyr121, Pro122, Phe125 and Val219. The common binding residues included Phe81, Trp84 and Leu85 [46].

*OPG*: The binding region for OPG, derived from the PDB complex with RANKL, extended from residue 31 to 186. Our literature search yielded a set of binding residues, namely Glu116, Phe117, Glu114, Thr55, Ser63, Pro64, Val65, Glu68, Glu93, Glu95, Arg90 and Phe96. Lastly, the consensus for OPG emphasized the importance of the region spanning from residue 31 to 186 in facilitating interactions [47] (Additional file 1: Table S2).

### Identification of interacting sites for ligands

Similarly, interacting sites for ligands of their respective receptors were identified through a comprehensive literature search, SiteHound, PDB and Active Site Prediction. The details of all identified IR residues are provided in Additional file 1: File S3.

*IL8*: The binding regions of IL8 with CXCR1 as identified from PDB complexes [44] spanned from residue 19 to 99. The literature search highlighted the significance of the N-domain, beta-sheet and C-terminal regions of IL8 for binding with CXCR1 [44]. A comparison of binding residues from all sources revealed common residues such as Tyr13, Phe17, Pro19, Lys20, Ile22, Lys23, Ile40, Pro46, Glu48, Leu49, Cys50 and Phe65 (Additional file 1: Table S3). The key binding residues included Thr12, Phe17, Lys20, Ser44, Pro46, Glu48, Leu49, Cys50 and Val61.

*TL1A*: The binding region identified from the PDB complex of DcR3 and TL1A, spanned from residue 72 to 251. The literature review revealed residues Arg36, Leu56, Gly57, Met91, Asp108, Ser120, Tyr121, Phe122, Glu123, Phe124, Asn140, Phe142, Thr172 and Glu174 for DcR3-TL1A binding [46]. A comparison of binding residues from all sources indicated that Ser120 is an essential residue in the binding site.

*RANKL*: The binding region established from the PDB complex of RANKL and OPG, covered residues 162 to 317. In literature, residues from 177–183 and 162–317 regions of RANKL, were reported as crucial for binding with OPG. Specific residues identified included Lys180, Asp189, Arg190, His223, Gln236 and Ile249 involved in binding [47]. A comparison of binding residues from all sources indicated that the region 177–183 is vital for receptor binding, and important residues such as His223, Gln236 and Thr182 were found consistent across all the sources (Additional file 1: Table S3).

### Selection of amino acid residues for peptide designing

Following the identification of consensus regions as detailed above, we selected specific amino acid residues to design peptides (Table 2).

*CXCR1-IL8:* The binding domain with residues HPKFIKELR (His18- Pro19- Lys20- Phe21- Ile22- Lys23- Glu24- Leu25-Arg26) is known to interact with the extracellular loop of its receptor CXCR1. This domain is of particular interest as its ELR (glutamic acid-leucine-arginine) motif plays a role in promoting angiogenesis when interacting with the CXCR1 receptor. Therefore, this domain was selected for designing a peptide with the goal of potentially inhibiting angiogenesis (Table 2).

*DcR3-TL1A:* DcR3 binds to the DE loop and AA loop of TL1A, from which important residues were identified. The first selected domain, TDSYPEP (Thr118-Asp119-Ser120-Tyr121-Pro122-Glu123-Pro124), was selected from the DE loop of TL1A. This selection was primarily based on the presence of critical interacting residues, Ser120 and Tyr121. The second domain was chosen from the AA loop with the sequence TKEDKTF (Thr172- Lys173- Glu174- Asp175- Lys176- Thr177- Phe178) because it contains residues Thr172 and Glu174, which are essential for binding to the DcR3 receptor. Additionally, another binding region within the AA loop with the sequence LGLAFTK (Leu56- Gly57- Leu58- Ala59- Phe60- Thr61- Lys62) was also selected for peptide design, as it contains Leu56 and Gly57, which are important for receptor binding (Table 2).

*OPG-RANKL:* The residue Lys248 forms a hydrogen bond with OPG, and Ile249 in the DE loop of RANKL provides specificity in the interaction with DcR3. Therefore, the region SIKIPSS (Ser246- Ile247- Lys248- Ile249- Pro250- Ser251- Ser252) was chosen for peptide design. Additionally, the residues Pro300, Asp301 and Gln302 of RANKL are involved in interaction with OPG. Therefore, the region PDQDATYP (Pro300-Asp301- Gln302- Asp303- Ala304-Thr305-Tyr306-Pro307) was selected for designing a peptide (Table 2).

### Peptide generation

Initially, 26 peptides were generated for CXCR1, 23 for DcR3 and 21 for OPG. The physicochemical properties of all the peptides were studied by AntiCP (Additional file 1: Table S4). Following analysis of the physicochemical properties of the peptide analogs using Antic, the top 5 candidates that represented the best anti-cancer peptides were selected (Table 3). Analog selection was based on analogs having properties similar to the reference peptides in AntiCP and they were subsequently subjected to docking studies with their respective TR.

**Table 3:** Physiochemical properties of selected peptides characterized using AntiCP

Target receptor	Peptides labels	Peptides sequence	HPo	HP	HPi	AP	Charge
CXCR	C9	HPKFELY	− 0.15	− 0.99	0.91	− 0.16	0.5
	C7	HPKFEWL	− 0.1	− 0.93	− 0.9	− 0.31	0.5
	C5	HPKFEWR	− 0.42	− 2.11	1.26	0.37	1.5
	C1	FHPKELY	− 0.15	− 0.99	0.91	− 0.16	0.5
	C26	HPKF	− 0.24	− 1.48	0	1.28	1.5
DcR3	D6	ADSYPQP	− 0.03	− 0.6	0.18	− 0.69	0
	D18	AFSYPFP	0.16	0.3	0	− 1.07	0
	D8	TDSYPAP	− 0.15	− 1.1	0	0.01	− 1
	D2	ADSYPEP	− 0.21	− 1.5	0.18	0.5	− 2
	D12	TDSYPFP	− 0.1	− 0.96	0	− 0.27	− 1
OPG	P10	HDQDATYF	− 0.23	− 1.39	0.34	0	− 1.5
	P5	KTSIKIPS	− 0.08	0.31	0.92	0.15	2
	P16	PDQDATYP	− 0.27	− 1.74	0.16	0.38	− 2
	P19	PDTYPQDP	− 0.31	− 2.16	0.16	0.44	− 2
	P8	CDQDATYF	− 0.17	− 0.68	0.16	− 0.06	− 2

HPo; hydrophobicity, HP: hydropathicity, HPi; hydrophilicity, AP; amphipathicity, molecular weight, charge and anticancer properties

### Comparison of docking energies

To assess the binding potential of the peptides, the peptides were docked with the TRs and binding energies were evaluated. The binding energies of various poses of shortlisted peptides are shown in Table 4. The complexes were then selected based on lower binding energy values, indicating greater stability. The binding energies of various poses of all the peptides are shown in Additional file 1: Table S5.

**Table 4:** Predicted docking energies of target proteins with selected peptides

Target	Peptide	Peptide	Docking energies and poses									
Receptor	Label	Sequence	1	2	3	4	5	6	7	8	9	FBE
CXCR	C9	HPKFELY	− 8.4	− 8.2	− 8.1	− 8	− 8	− 8	− 7.8	− 7.5	− 7.3	− 8.4
	C7	HPKFEWL	− 8.3	− 8	− 7.9	− 7.9	− 7.8	− 7.7	−	−	− 7.5	− 8.3
	C5	HPKFEWR	− 8.1	− 8.1	− 8.1	− 7.9	− 7.8	− 7.6	−	−	− 7.1	− 8.1
	C1	FHPKELY	− 8.1	− 8.1	− 8	− 7.8	− 7.7	− 7.7	− 7.7	− 7.6	− 7.6	− 8.1
	C26	HPKF	− 4.7	− 4.3	− 4.2	− 4.2	− 4.2	− 4.1	− 4	− 3.9	− 3.7	− 4.7
DcR3	D6	ADSYPQP	− 7.2	− 6.3	− 6.1	− 6.1	− 6	− 6	− 6	− 6	− 5.9	− 7.2
	D18	AFSYPFP	− 7.2	− 7.1	− 7.1	− 6.9	− 6.8	− 6.8	− 6.8	− 6.7	− 6.7	− 7.2
	D8	TDSYPAP	− 6.7	− 6.7	− 6.5	− 6.5	− 6.4	− 6.4	− 6.4	− 6.3	− 6.3	− 6.7
	D2	ADSYPEP	− 6.4	− 6.2	− 6	− 5.9	− 5.9	− 5.9	− 5.9	− 5.8	− 5.8	− 6.4
	D12	TDSYPFP	− 5.8	− 5.7	− 5.6	− 5.6	− 5.6	− 5.6	− 5.6	− 5.5	− 5.5	− 6.8
OPG	P10	HDQDATYF	− 6.6	− 6.5	− 6.3	− 6.2	− 6.2	− 6.1	− 6.1	− 6.1	− 6	− 6.6
	P5	KTSIKIPS	− 6.6	− 6.1	− 6.1	− 6	− 6	− 6	− 5.9	− 5.9	− 5.9	− 6.6
	P16	PDQDATYP	− 6.8	− 6.3	− 6.3	− 6.3	− 6.2	− 6.1	− 6.1	− 6.1	− 6	− 6.8
	P19	PDTYPQDP	− 6.9	− 6.8	− 6.8	− 6.8	− 6.6	− 6.6	− 6.5	− 6.5	− 6.4	− 6.9
	P8	CDQDATYF	− 6.5	− 6.4	− 6.2	− 6.1	− 6.1	− 6	− 6	− 6	− 5.9	− 6.5

*FBE* Final Binding Energy

For CXCR1, the binding energies of the designed peptides ranged from − 8.4 to − 6.4. The peptide C9 exhibited the best binding affinity with a binding energy of − 8.4. Additionally, C7 bound with a binding energy of − 8.3, while C1 and C5 showed binding energies of − 8.1. The peptides D1, D6 and D18 exhibited the most favorable binding energies of − 7.2 against DcR3, whereas D7 displayed the least binding energy at − 4.7. In the case of OPG, peptide P19 demonstrated a strong binding energy of − 6.9, surpassing the other peptides (Table 4). Furthermore, peptides P16, P5, P10, P8, P20, P6, P7 and P18 exhibited moderate binding energies ranging from − 6.0 to − 6.8 (Additional file 1: Table S5).

### Post-dock interaction analysis

**Fig. 3:**
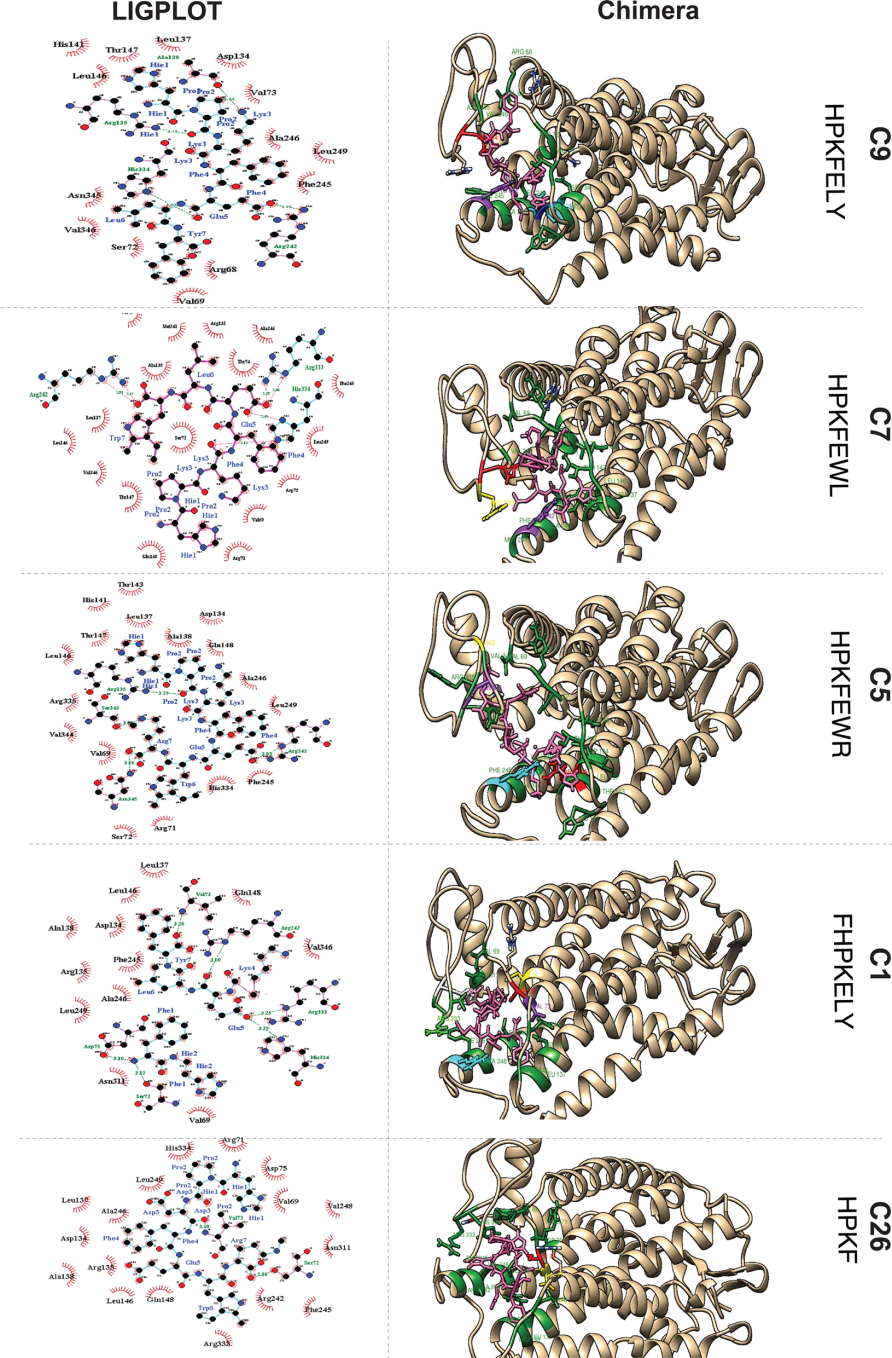
Post-Docking Interaction of CXCR1 with Respective Peptides. *Left Panel:* Schematic Representation using LIGPLOT. Pink lines represent non-ligand bonds. Cyan lines denote ligand bonds. Green dotted lines, with annotated distances, indicate hydrogen bonds. Red hemispheres signify hydrophobic interactions. *Right Panel:* Visualization in Chimera. All hydrophobic residues are depicted in green. The ligands are consistently represented in pink in all interactions. C9: His334 (red), Arg135 (cyan), Ala138 (yellow), and Arg242 (purple) form hydrogen bonds. C7: His334 (red), Arg333 (yellow), and Arg242 (purple) form hydrogen bonds. C5: Arg135 (red), Arg242 (yellow), Ser343 (purple), and Asn345 (cyan) form hydrogen bonds. C1: Ser72 (red), Val73 (purple), Asp75 (yellow), Arg242 (cyan), Arg333 (light green) and His334 (blue) form hydrogen bonds. C26: Ser72 (red) and Val73 (yellow) form a hydrogen bond

*Post-Docking Interaction Analysis of CXCR1 and Peptides: The* post-docking interaction of CXCR1 with C9, C7, C5, C1 and C26 is shown in Fig. 3. The interaction analysis of peptides with the target protein CXCR1 reveals crucial details about the binding interactions (Additional file 1: Table S6).

*Peptide C9:* The post-docking interaction of CXCR1 with peptide C9 is shown in Fig. 3. Amino acid residues Arg135, Ala138, Arg242 and His334 of CXCR1 form hydrogen bonds with Phe4, Glu5, Lys3 and Pro2 of peptide C9. Arg68, Val69, Val73, Ser72, Asp134, Leu137, His141, Leu146, Thr147, Phe245, Ala246, Leu249, Asn345 and Val346 of CXCR1 engage in hydrophobic interactions with peptide C9. The pink lines show non-ligand bonds, cyan lines show ligand bond and green dotted lines with distances mentioned show hydrogen bond and red hemispheres shows hydrophobic interactions. Interactions are visualized in Chimera and the ligand is colored pink His334, Arg135, Ala138 and Arg242 are colored red, cyan, yellow and purple respectively, indicating their formation of hydrogen bonds. The hydrophobic residues are represented in green color.

*Peptide C7:* The residues of CXCR1 including Arg242, Arg333 and His334 form hydrogen bonds with peptide C7. Amino acids Val69, Arg71, Ser72, Thr74, Asp75, Arg135, Leu137, Ala138, Leu146, Thr147, Gln148, Met241, Phe245, Ala246, Leu249 and Val346 of CXCR1 participate in hydrophobic interactions with peptide C7.

*Peptide C5:* The residues Arg135, Arg242, Ser343 and Asn345 of CXCR1 establish hydrogen bonds with Pro2, Arg7 and Glu5 residues of peptide C5. Val69, Arg71, Ser72, Asp134, Arg135, Leu137, Ala138, His141, Thr143, Leu146, Thr147, Gln148, Fhe245, Ala246, Val248, Leu249, His334, Arg335 and Val344 of CXCR1 engage in hydrophobic interactions with peptide C5.

*Peptide C1 and C26*: Ser72 and Asp75 of CXCR1 form hydrogen bonds with residues Phe1 and Val73 of peptide C1. Val73 and Arg242 of CXCR1 form hydrogen bonds with Tyr7 and Lys4 of peptide C1. Arg333 and His334 of CXCR1 form hydrogen bonds with Glu5 of peptide C1. Ser72 and Val73 of peptide C26 establish hydrogen bonds with Arg7 and Asp3 of CXCR1. Val69, Arg71, Asp75, Asp134, Arg135, Leu137, Ala138, Leu146, Gln148, Arg242, Phe245, Ala246, Val248, Leu249, Asn311, Arg333 and His334 of CXCR1 participate in hydrophobic interactions with these peptides.

These detailed interaction analyses provide insights into the binding mechanisms of the peptides with CXCR1, shedding light on the specific amino acid residues involved in hydrogen bonding and hydrophobic interactions, which are critical for understanding the binding affinity and potential therapeutic applications.

**Fig. 4:**
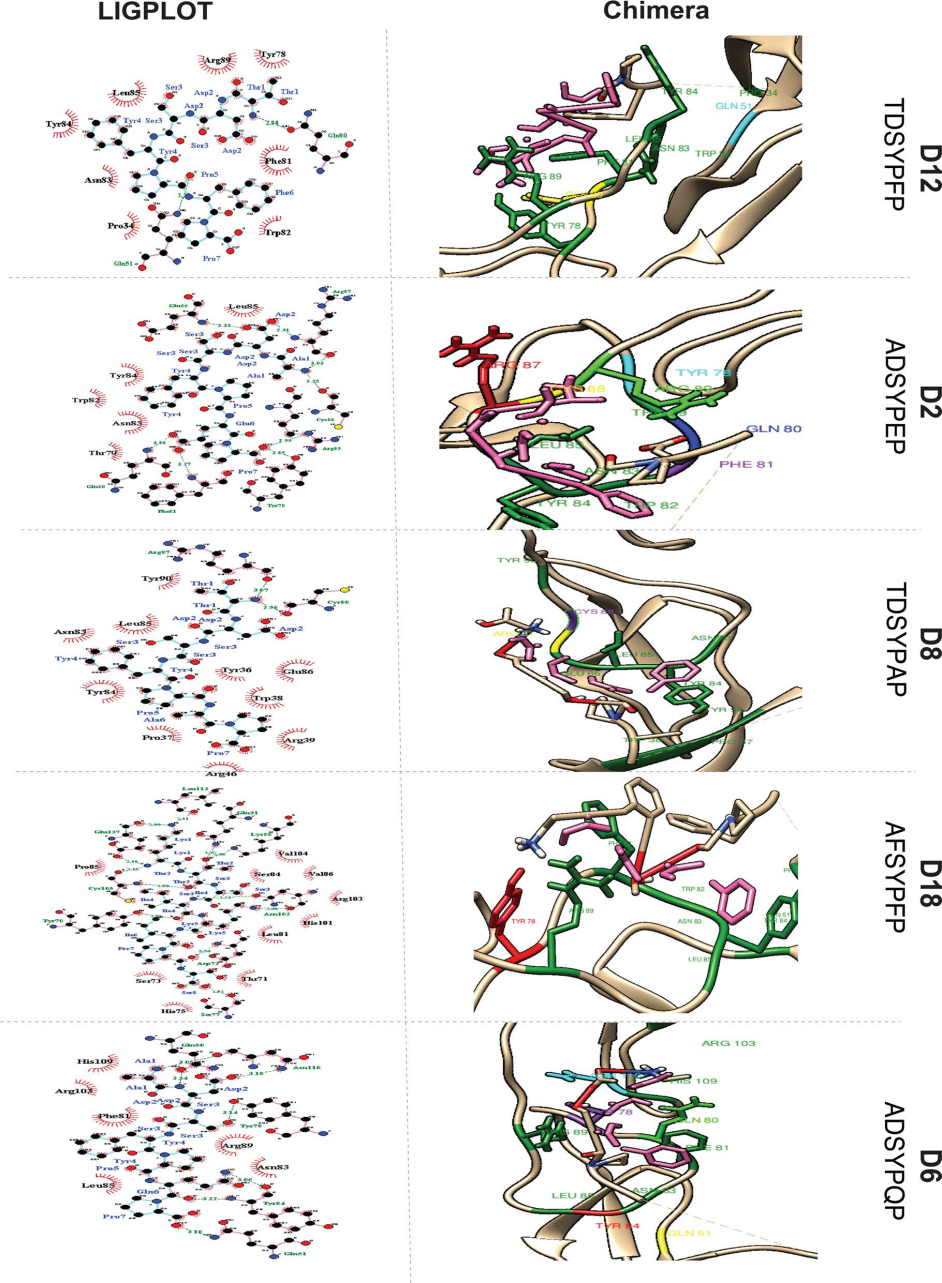
Post-Docking Interaction of DcR3 with Respective Peptides. *First Panel:* Schematic Representation of LIGPLOT view. Pink lines represent non-ligand bonds. Cyan lines denote ligand bonds. Green dotted lines, with annotated distances, indicate hydrogen bonds. Red hemispheres signify hydrophobic interactions. *Second Panel:* Visualization in Chimera. All hydrophobic residues are colored green. The ligands are consistently represented in pink in all interactions. D6: Gln51 (yellow), Tyr78 (purple), Gln80 (light green), and Tyr84 (red) of DcR3 each form one hydrogen bond with the peptide. Asn110 (cyan) forms two hydrogen bonds with the peptide. D18: Tyr78 (red) forms one hydrogen bond with the peptide. D8: Arg87 (yellow) and Cys88 (purple) each form one hydrogen bond with the peptide. D2: Tyr78 (cyan), Gln80 (blue), Phe81 (purple), Glu86 (light green) and Cys88 (yellow) of DcR3 form single hydrogen bonds with Pro7, Glu6, Glu6, Asp2, and Ala1 of peptide D2, respectively. Arg87 forms two hydrogen bonds with Asp2 and Ala1. D12: Gln51 (cyan) and Gln80 (yellow) of DcR3 form single hydrogen bonds with Pro5 and Thr1, respectively

*Post-Dock Interaction Analysis of DcR3 and Peptides:* The post-docking interaction of DcR3 with D6, D18, D8, D2 and D12 is shown in Fig. 4 and Additional file 1: Table S6. The analysis of interactions between peptides and the DcR3 receptor provides insights into the binding mechanisms.

*Peptide D6:* The residues Gln51, Tyr78, Gln80 and Tyr84 of DcR3 form hydrogen bonds with Pro7, Ser3, Ala1 and Gln6 of peptide D6. Asn110 of DcR3 forms two hydrogen bonds with residues Asp2 and Ala1 of the DcR3 receptor. The residues Phe81, Asn83, Leu85, Arg89, Arg103 and His109 of DcR3 engage in hydrophobic interactions.

*Peptide D18:* In the docked complex of DcR3 and D18, only one hydrogen bond is formed by Tyr78 of DcR3 with Ala1 of the peptide. The hydrophobic interactions are formed by Pro34, Gln51, Phe81, Trp82, Asn83, Tyr84, Leu85 and Arg89 of DcR3.

*Peptide D8:* The residues Arg87 and Cys88 of DcR3 both form a hydrogen bond with residue Thr1 of peptide D8. Hydrophobic interactions are formed by Pro37, Trp38, Arg39, Arg46, Asn83, Tyr84, Leu85 and Tyr90 of DcR3.

*Peptide D2:* The residues Tyr78, Gln80, Phe81, Glu86 and Cys88 of DcR3 form single hydrogen bonds with Pro7, Glu6, Glu6, Asp2 and Ala1 of peptide D2. Arg87 forms two hydrogen bonds with Asp2 and Ala1. The hydrophobic interactions are formed by Thr79, Trp82, Asn83, Tyr84 and Leu85 of DcR3.

*Peptide D12:* The residues Gln51 and Gln80 of DcR3 form hydrogen bonds with Pro5 and Thr1 residues of peptide D12. The residues Pro34, Tyr78, Phe81, Trp82, Asn83, Tyr84, Leu85 and Arg89 of DcR3 engage in hydrophobic interactions.

These detailed interaction analyses provide critical insights into the binding modes of the peptides with the DcR3 receptor. Understanding the specific amino acid residues involved in hydrogen bonding and hydrophobic interactions is essential for assessing the binding affinity and potential therapeutic applications.

**Fig. 5:**
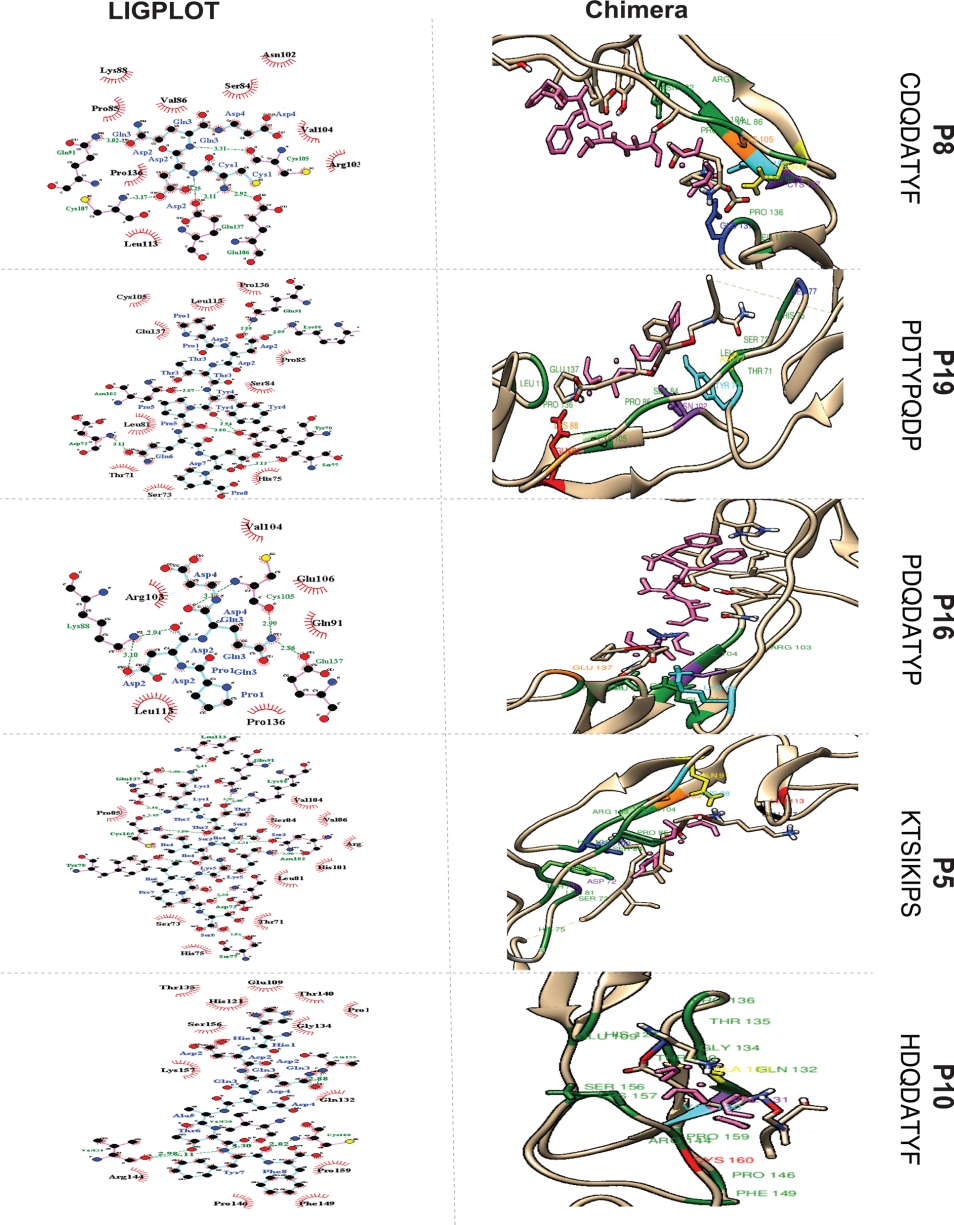
Post-Docking Interaction of OPG with Respective Peptides. *Left Panel:* Schematic Representation using LIGPLOT. Pink lines denote non-ligand bonds. Cyan lines indicate ligand bonds. Green dotted lines with annotated distances represent hydrogen bonds. Red hemispheres depict hydrophobic interactions. *Right Panel* shows a visualization of a chimera. All hydrophobic residues are represented in green. The ligands are colored pink in all interactions. P10: Residues Val130 (cyan), Val131 (purple), Ala133 (yellow), and Cys160 (red) of OPG form single hydrogen bonds. P5: Residues Tyr70 (light green), Asp72 (purple), Ser77 (white), Gln91 (yellow), Lys88 (cyan), Asn102 (blue), Cys105 (orange), Leu113 (red), and Glu1437 (black) of OPG form single hydrogen bonds. P16: Glu137 (orange) forms a single hydrogen bond with Gln3. Cys105 (purple) forms two hydrogen bonds with Gln3 and Asp4. Lys88 (cyan) forms two hydrogen bonds with Asp2. P19: Asp72 (yellow), Ser77 (blue) and Asn102 (purple) form single hydrogen bonds with Gln6, Asp7, Tyr4. Lys88 (orange) and Gln91 (red) form single hydrogen bonds with Asp2. Tyr70 (cyan) forms two hydrogen bonds with Tyr4 and Pro5. P8: Gln91 (yellow) and Cys105 (orange) form a single hydrogen bond with Gln3. Glu106 (cyan) forms a single hydrogen bond with Cys1. Glu137 (blue) forms two hydrogen bonds with Cys1 and Asp2

*Post-Dock Interaction Analysis of OPG and Peptides:* The post-docking interaction of OPG with P10, P5, P16, P19 and P8 is shown in Fig. 5 and Additional file 1: Table 6. Furthermore detailed interaction analysis between peptides and the OPG receptor provides essential insights into the binding mechanisms.

*Peptide P10:* The residues Val130, Ala133, and Cys160 of OPG form single hydrogen bonds with Tyr7, Gln3 and Phe8 of peptide P10. Val131 forms two hydrogen bonds with Thr6 and Tyr7 of the peptide. The hydrophobic interactions are formed by Glu109, His121, Gln132, Gly134, Thr135, Pro136, Thr140, Arg144, Pro146, Phe149, Ser156, Lys157 and Pro159 of OPG.

*Peptide P5*: The residues Thy70, Asp72, Ser77 and Leu113 of OPG form single hydrogen bonds with Ile4, Ser8, Ser8, Thr2 and Lys1. Lys88 and Gln91 form hydrogen bonds with Thr2 of P5. Cys105 forms hydrogen bonds with Lys1 and Thr2, Asn102 forms a hydrogen bond with Ser3 and Lys5, and Glu137 forms two hydrogen bonds with Lys1. The hydrophobic interactions involve Thr71, Sr73, His75, Leu81, Ser84, Pro85, Val86, His101, Arg103 and Val104 of OPG.

*Peptide P16:* Glu137 forms a single hydrogen bond with Gln3 while Cys105 forms two hydrogen bonds with Gln3 and Asp4. Lys88 forms two hydrogen bonds with Asp2 of P16. The hydrophobic interactions involve Gln91, Arg103, Val104, Glu106, Leu113 and Pro136.

*Peptide P19:* Residues Asn102, Asp72, Ser77, Tyr7 and Lys88 form a single hydrogen bond with Tyr4, Gln6, Asp7, Tyr4, Asp2 and Asp2 respectively. The hydrophobic interacting residues of OPG include Ser84, Pro85, Thr71, Ser73, His75, Leu81, Cys105, Leu113, Pro136 and Gln137. The residues Gln91 and Cys105 form a single hydrogen bond with Gln3, Glu106 forms a single hydrogen bond with Cys1 and Glu137 forms two hydrogen bonds with Cys1 and Asp2 of P8.

These detailed interaction analyses provide critical insights into the binding modes of the peptides with the OPG receptor. Understanding the specific amino acid residues involved in hydrogen bonding and hydrophobic interactions is essential for assessing the binding affinity and potential therapeutic applications.

### Homology finding

In addition to interacting with the target receptors, it is important to consider that they can also bind with other receptors. Besides CXCR1, IL8 also binds with other receptors, namely CXCR2 and DARC (Duffy Antigen Receptor Chemokine). The homology between the N-domain of CXCR1 and CXCR2 is 27.1%. The homology between the N-domains of CXCR1 and DARC is 20.3%. Similarly, TL1A binds with the TNFRSF25 protein (Tumor Necrosis Factor Receptor Superfamily-25), which has binding regions for TL1A. The homology between DcR3 and TNFRSF25 is 16.3%. RANKL exhibited a low binding affinity for homologs of OPG, i.e., 0.5% and 15.6% for DR5 and RANK, respectively.

### Docking of peptides with other receptors of ligands

The docking results of shortlisted peptides against homologues of CXCR1, DcR3 and OPG are summarized in Additional file 1: Table S7. The peptides C9, C7, C5, C1 and C26 for CXCR1 were docked with its homologues CXCR2 and DARC. These simulations revealed a higher binding affinity with DARC compared to CXCR2. The peptides designed for DcR3 were also docked with TNFRSF25 and TNFRSF21, resulting in binding energies ranging from − 6.0 to − 5.3 for TNFRSF25 and − 6.1 to − 5.2 for TNFRSF21, indicating relatively low binding affinity compared to DcR3. The peptides for OPG were docked with RANK and DR5, showing relatively low binding affinity with both. Overall, our results suggest that the selected peptides exhibited a higher degree of binding affinity with the target receptors compared to their homologues.

## Discussion

Recent literature recognizes the significance and cost-effectiveness of *in-silico tools* and computational models in designing novel anticancer peptides [36, 48, 49]. These peptides exhibit significant potential in selectively binding to differentially expressed cell surface receptors and proteins, including immune checkpoints, receptor kinases, and hormone receptors in cancer, thereby effectively inhibiting their biological activity [49]. Moreover, compared to other larger molecules such as monoclonal antibodies, peptides possess a better ability to penetrate cell membranes and disrupt protein–protein interactions with intracellular proteins [48–50, 48, 51]There is compelling evidence that peptide-based therapeutics have emerged as promising agents in cancer treatment, offering opportunities for the development of novel peptides.

Decoy receptors such as CXCR or DcR are emerging as actionable targets that can potentially be blocked by therapeutic drug candidates to suppress oncogenic signaling [6, 51]. CXCR1, in association with its ligand IL8, governs leukocyte recruitment into tumor cells and influences the tumor immune response. It [52] also regulates angiogenesis, promotes tumor growth and survival, and facilitates metastasis [52]. Several peptide decoys, including cell-penetrating decoy peptides, TNRF-ECD, and HIRMAb-TNFR fusion protein, have been developed to inhibit the interaction of chemokine receptor signaling and are being assessed as therapeutic agents [6] Recently, Chang et al. [53] developed an antagonist peptide using similar approach, aimed at CXCR1/2, to impede downstream signaling pathways by competitively binding with IL-8 at CXCR1/2 sites. RF16 demonstrated efficacy in diminishing cell proliferation, migration, and invasiveness in MDA-MB-231 cells.

Our research theoretically proposes potential peptides with high affinity for decoy receptors such as CXCR1, DcR3 and DARC, the latter of which binds to angiogenic chemokines. Consequently, these peptides hold promise as robust therapeutic candidates. The strong binding affinity of these peptides with these receptors suggests their potential to inhibit apoptosis while simultaneously inhibiting angiogenesis through their neutralizing effect on DARC. This dual action may effectively impede both apoptosis and angiogenesis [46, 54].

Osteoprotegerin (OPG) plays a key role in regulating bone metastasis, controlling tumor invasion within bone tissue, and modulating cellular integrity [54]. The OPG/RANKL/RANK pathway holds a pivotal role in bone homeostasis and represents a therapeutic target for various bone diseases, including osteoporosis [55, 56]. OPG, as a soluble decoy receptor, binds to RANKL and inhibits its interaction with RANK, effectively preventing osteoclastogenesis and bone resorption [55]. In a previous study [57], peptides derived from OPG-RANKL interaction demonstrated efficacy in inhibiting RANKL, thereby mitigating bone loss while preserving inflammatory processes. In this study, Leu113-Arg122 was identified as a putative site for peptide synthesis. Building upon the OPG-RANKL interaction, our study proposed two regions, SIKIPSS and PDQDATYP, as templates for peptide design.

The peptides designed in this study are not only highly specific but also mimic the natural ligands of the targeted receptors. Due to their potential for highly specific binding and low expected immunogenicity, these peptides are promising candidates for impeding ligand-receptor interactions and hindering downstream signaling pathways. However, certain limitations, such as the inability to perform MD simulation due to resource constraints, are acknowledged. To fortify the study, it is imperative to undertake experimental validation and further optimize the designed peptides to validate their potential as targeted anti-cancer therapies. Additionally, an in-depth exploration into the specificity and selectivity of the peptides concerning cancer cells versus normal cells will be conducted to assess their therapeutic potential. Furthermore, the optimization of peptide sequences through iterative design modifications will be conducted based on the outcomes of experimental validation. This iterative process entails refining the peptide structures to improve binding affinity, specificity and stability.

## Conclusions

This study presents a strategy for designing peptides targeting receptors commonly overexpressed in cancers using *in-silico* methods and computational methods. The approach capitalizes on the interaction between receptors and their native ligands to enhance target specificity, ensuring a more precise and targeted impact. By mimicking natural ligands, the peptides aim to minimize the immunogenic response, thereby making the approach more clinically favorable. Following interaction analysis, peptides targeting CXCR1, DcR3 and OPG have been shortlisted and will be further pursued for in-vivo experiments.

### Supplementary Information

**Additional file 1: Table S1.** The physiochemical properties of OPG obtained from Protparam. **Table S2.** Comparison of interactive residues in target receptors identified from prediction software, PDB and literature search. **Table S3.** Comparison of Interacting residues in ligands identified from prediction software, PDB and literature search. **Table S4.** Physiochemical properties of the designed peptide library for CXCR1, DcR3 and OPG. **Table S5.** Comparison of docking energies for different poses of peptides against their target receptors. **Table 6.** The post-dock interactions analysis of target receptors and their respective peptides. **Table S7.** Docking results of shortlisted peptides of CXCR1, DcR3 and OPG with their respective homologous proteins.

## Data Availability

Not applicable.
